# Myco-Facilitated Biosynthesis of Nano-Silver From Wasp Nest Fungus, *Paecilomyces variotii*, and Its Antimicrobial Activity Against MTCC Strains

**DOI:** 10.3389/fmicb.2022.841666

**Published:** 2022-04-06

**Authors:** B. K. Nayak, V. Prabhakar, Anima Nanda

**Affiliations:** ^1^Department of Botany, Kanchi Mamunivar Government Institute for Post Graduate Studies and Research (Autonomous), Puducherry, India; ^2^Department of Biomedical Engineering, Sathyabama Institute of Science and Technology, Chennai, India

**Keywords:** nano silver, wasp nest fungus, *Paecilomyces variotii*, antimicrobial activity, MTCC strains

## Abstract

The utility of fungi as stabilizing and reducing agents in the biogenic synthesis of silver nanoparticles is striking due to the production of large quantities of biomolecules of minute toxic residuals. During the current study, sunlight- and dark-assessed silver nanoparticles were synthesized from wasp nest fungus, *Paecilomyces variotii*, at different pHs. Synthesized silver nanoparticles (AgNPs) at 6 pH were found to be more prominent than at 7 and 8 pHs. AgNPs were within the 20- to 90-nm range and were polygonal and elongated in shape. FTIR spectra of light-mediated AgNPs showed diverse transmittance bands than the silver nanoparticles synthesized in the dark. The synthesized AgNPs were found with diverse antimicrobial activities against pathogenic MTCC bacterial strains, i.e., *Staphylococcus aureus*, *Vibrio parahaemolyticus*, *Escherichia coli*, *Shewanella putrefaciens*, and fungus, *Candida albicans*. Aqueous filtrate and filtrate-mediated AgNPs combined with methanol solvent extract of yeast extract manitol broth (YEMB) had more inhibitory effects on all bacteria and *Candida albicans*. Furthermore, the combined effect of AgNPs and methanol solvent extract from YEMB culture filtrate was found more effective against *E. coli*, while AgNPs combined with methanol solvent of aqueous filtrate had inhibitory effects on *E. coli* and *Candida albicans.*

## Introduction

Microbes are considered as important sources of bioactive natural products with enormous potential for the invention of new biomolecules for drug breakthrough, industrial utility, and agricultural applications (Scott et al., 2008; Usha et al., 2010; Devaraj et al., 2013; Nayak et al., 2018a). The earlier studies based on estimation of microbial populations have given away that only about 1% of bacteria and 5% of fungi are characterized and the rest remain uncharted for their contribution to human welfare (Usha et al., 2010; Nayak et al., 2018a). In recent years, the microbes have become resistant to the drugs and are subsequently modifying their genomic sequence. It becomes a new challenge for the researchers to look for efficient drugs in order to restrict the drug-resistant microbes (Demain, 1999; Lalitha, 2013; Misra et al., 2020, 2021). A case study by World Health Organization showed that 90% of the bacterial strains are found to be resistant to drugs. Drug resistance in bacteria has become a global concern, and the search for new antibacterial agents is urgent and ongoing (Dhamodharan et al., 2017). Traditional sources to discover novel antibiotics appear to be largely shattered (Saravanan et al., 2007; Honary et al., 2013). In contempt to bioprospecting soil Actinomycetes, fungi, the most significant source of new antibiotics in the 20th century results in the rediscovery of known compounds (Saravanan et al., 2007; Honary et al., 2013; Gudikandula et al., 2017). A further approach is identifying sources of microbes that have not been explored for their potential natural commodities (Nayak et al., 1998, 2013, 2018b; Demain, 1999; Saravanan et al., 2007; Fischbach and Walsh, 2009; Prakash and Thiagarajan, 2012; Sagar and Ashok, 2012; Nayak and Anitha, 2014). Symbiotic microbes may represent a particularly promising source because microbial symbioses are widespread, widely unexplored for natural products (Demain, 1999; Prakash and Thiagarajan, 2012) and often involve the exchange of small molecules between symbionts and host including compounds mediating host defense (Sagar and Ashok, 2012; Nayak and Anitha, 2014; Bhat et al., 2015; Nayak et al., 2018b). Among symbiotic associations, insect–microbe symbioses that involve fungi may be of particular interest in natural product discovery (Sagar and Ashok, 2012; Nayak et al., 2018a).

By looking at the recent strategies in addressing the urgent need for new antibiotics, researchers have used the symbiotic relation of wasp and fungi, where both are well known for protecting the host. On the other hand, scientists have also used the novel compound into nanoparticles in enhancing the effectiveness toward multidrug resistance organisms (Strobel, 2006; Hany et al., 2014; Majeed et al., 2014). Biological synthesis of metallic nanoparticles using various living systems, such as fungi, bacteria, algae, and plant extract have been reported (Lara, 2010; Nayak et al., 2014a,b; Zarina and Nanda, 2014a). Several studies have been reported on the antimicrobial effects of AgNPs mainly using laboratory strains or pathogenic microbes. However, the antibacterial and antifungal mechanisms of biologically synthesized AgNPs have not been elucidated thoroughly. Therefore, AgNPs are only known to inhibit bacterial growth by cell membrane attachment, penetration, and release into the organisms (Nayak et al., 2014b). Currently, scientists have shown unexpected interest in biomaterial therapeutic agents to overcome the problem of drug resistance among bacteria caused by the disoperation of antibiotics (Nanda and Majeed, 2013). In this study, the wasp nest soil fungi were selected for biosynthesis of silver nanoparticles. Very few studies have been reported that isolate fungi and other microorganisms like bacteria and actinobacteria from wasp nest soil. Hence, our experiment was made to screen and isolate fungi from wasp nest soil and to synthesize silver nanoparticles by extracellular method in light and dark conditions. The myco-synthesized silver nanoparticles were further subjected to characterization and antimicrobial assay against MTTC pathogenic bacteria and *Candida albicans* procured from IMTECH, Chandigarh, India.

## Materials and Methods

### Isolation of Wasp Nest Fungi

Empty wasp nests were collected from Muthu Pillai Palayam village of Puducherry Dist. Puducherry state. Fungi were isolated and enumerated by serial dilution method from wasp nest soil (Saravanan et al., 2007; Johnson, 2014). Microbial suspension (1 ml) of each dilution was added to the sterilized glass Petri plates, then potato dextrose agar (PDA) medium was added (pour plate method, triplicate in each dilution). Both, the sample solution and PDA were thoroughly mixed by gradually rotating the Petri plates in clockwise and counter clockwise directions. The plates were allowed to solidify for a few hours then sealed by paraffin paper and kept in a BOD incubator at 25 ± 3°C.

After 3 days of incubation, fungal colonies appeared on the surface of the culture plates. Each fungal colony was aseptically isolated and transferred to PDA slants from the mixed culture. Each fungal strain was identified by microscopically observing its colony features viz., color, texture, and also reverse color, etc., with conidia, conidiophores, and arrangement of spores with the help of the mycology expertise of the authors, available manuals, and textbooks. Among the isolated wasp nest soil fungi, *Paecilomyces variotii* was found with notable characteristics, such as high proliferation, producing yellow color to black color droplets over the mycelia surface, and pigment secretion was observed on the reverse side. Therefore, *P. variotii* was selected for the biosynthesis of silver nanoparticles.

### Isolation and Separation of Fungal Extracts (Culture Filtrate and Aqueous Filtrate)

The silver nanoparticle synthesis was carried out from the fungal isolate, *Paecilomyces variotii*. The fungal inoculum was transferred to three different 500-ml Erlenmeyer flasks each containing 250 ml of sterilized potato dextrose broth (PDB) at different pH viz., 6, 7, and 8, respectively, and underwent separately for the synthesis of AgNPs. The culture flask was incubated at laboratory room temperature (25°C ± 2°C) and was manually shaken at definite periods of interval per 24 h up to 7 day.

After 7 days of incubation, the fungal extract was filtered by Whatman filter paper No. 1 and the filtrate was kept in the refrigerator at 4°C. The fungal biomass was still kept in the culture flask where it was grown. Furthermore, it was washed with milli Q water at least three to five times to remove excess medium components. The fungal biomass weight was recorded, and then double distilled water (DDW) was added at 1:10 ratio (1 g biomass: 10 ml of DDW) and incubated at the same position for 72 h. Again, the fungal filtrate was filtered by using Whatman filter paper No. 1; this filtrate was considered as an aqueous filtrate, which was kept for further use.

### Preparation of Silver Nanoparticles

For the biosynthesis of AgNPs, 1 mM of concentrated AgNO_3_ stock solution was prepared; 1 ml of AgNO_3_ was added to 9 ml of culture filtrate/aqueous filtrate, separately. This sample solution was considered as a reaction mixture. These reaction mixtures were split into two sets. From the two sets, one set of reaction mixture was directly exposed under bright sunlight for a few minutes for photochemical reaction. The other set was kept in the dark for 72 h at laboratory conditions by warping with aluminum foil over the test tubes to avoid photochemical reactions during the experiment. The light-exposed sample was immediately centrifuged after color change was observed, and the dark-kept samples were also centrifuged after 72 h of incubation period. Both reaction mixture samples were centrifuged at 10,000 rpm for 30 min thrice, and the pellets were collected for further characterization.

### Characterization of Silver Nanoparticles

After centrifugation, the pellets were collected in powder form. This powder of AgNPs was again suspended in 1 ml of double distilled water. The synthesized AgNPs in aqueous solution was examined at different instrumental analysis.

### UV-Vis Spectrophotometer

Change in color of the reaction mixture was visually observed over a period of time. Absorption measurements of synthesized AgNPs in an aqueous solution of different samples were carried out after 72 h, using UV-Visible Spectrophotometric (Systronic 2201) analysis in the range of 350–700 nm (Nayak et al., 2014b). The surface plasmon resonance absorption peaks were observed and recorded. The synthesized silver nanoparticles were kept for a few months to check their stability.

### Scanning Electron Microscopy Analysis

The morphological shape and size of the synthesized nanoparticle were confirmed by scanning electron microscopy (HITACHI S-3400N, Puducherry University, Puducherry, India). The image was considered for the detection of particle size, and the three-dimensional image showed the average roughness and in-homogeneity of the cluster formation of nanoparticles.

### Fourier-Transform Infrared

The FTIR spectrum of AgNP samples was recorded on an FTIR instrument (Thermo-Nicolet 6700, Puducherry University, Puducherry, India). All dimensions were carried out in the range of 500–4,000 cm^–1^ at a resolution of 4 cm^–1^.

### X-ray Diffraction Analysis

The XRD analysis was utilized to determine the metallic nature, crystallinity, and face-centered cubic structure of silver nanoparticles. For analysis of XRD, the sample was prepared by centrifugation of the silver nanoparticle solution at 15,000 rpm for 20 min. The supernatant was discarded, and the pellet was washed with Milli-Q water three to four times and then dried in glass plates. The powder form of the sample was subjected for XRD analysis at the International Research Centre, Sathyabama University, Chennai, Tamil Nadu, India.

### Antimicrobial Study

The efficacy of antimicrobial assay of silver nanoparticles obtained from the wasp nest isolate fungus *Paecilomyces variotii* was tested against MTCC microbial pathogenic strains acquired from the Institute of Microbial Technology (IMTECH), Chandigarh, India *viz*., (*Staphylococcus aureus*-MTCC 6908; *Vibrio parahaemolyticus*-MTCC 451; *E. Coli*-MTCC 3222; *Shewanella putrefaciens*-MTCC 8104), and fungus (*Candida albicans*-MTCC 183) by the well diffusion method (Saravanan et al., 2007; Johnson, 2014). The pure culture of *Paecilomyces variotii* was grown with different pH, the fungal culture filtrate and aqueous filtrate were taken as bioactive compounds from the fungus of both the soils and were studied for its antimicrobial activity. The fungal filtrates underwent extracellular biosynthesis of silver nanoparticles *in vitro* with different pH. The bacterial and fungal test organisms were inoculated in nutrient broth and potato dextrose broth, respectively, for 12 h. The plates contain microbial culture, and each well was loaded with 30 μl (30 μg) of a concentration of silver nanoparticles. Furthermore, to check the synergistic effect of silver nanoparticles, the nanoparticles were loaded with 2 mg in 1 ml (30 μl/60 μg) of concentrated methanol extract from the culture filtrate of *Paecilomyces variotii* suspension and loaded to each well. The overnight-grown bacterial and fungal culture was plated on nutrient agar for the bacteria and potato dextrose agar for the fungus, respectively, by using cotton swabs. Wells were cut on the agar plates using a cork borer, and 30 μl of AgNP solution was dispensed in each well. The plates were incubated at 25°C ± 2°C for 24 h and then observed for the presence of zones of inhibition, which appeared as a clear area around the wells. Inhibition zone diameter was measured in millimeter by using a Himedia zone scale. The diameter (mm) of the inhibition zone was recorded. The agar plates (nutrient agar for bacteria and potato dextrose agar for fungus) were examined for the zone of inhibition, and the inhibition zone diameter was measured in millimeter (mm) and plotted on the tables and graphs.

## Results and Discussion

### Isolation of *Paecilomyces variotii*

**FIGURE 1:**
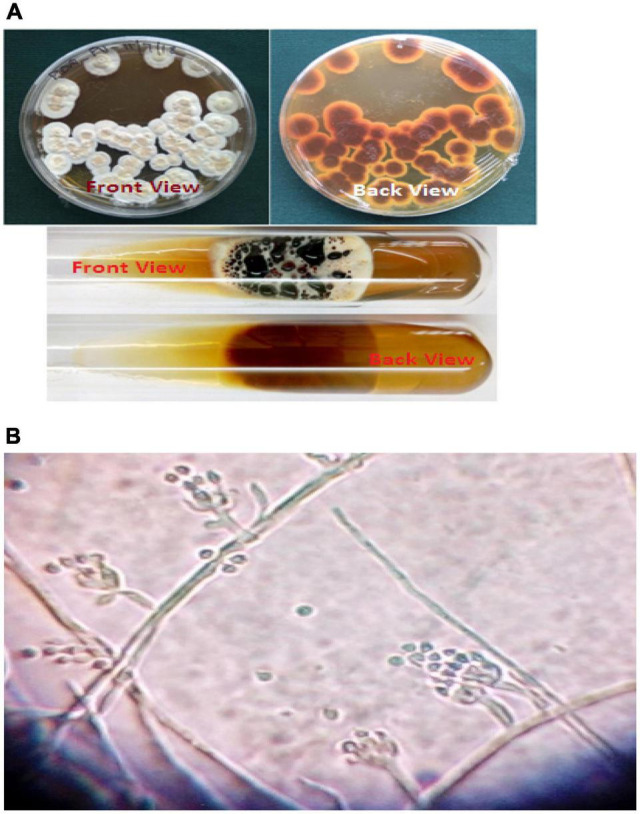
**(A)** Growth of *Paecilomyces variotii* on agar plate and tube cultures. **(B)** Microscopic view *of Paecilomyces variotii* (15 × 40×).

**FIGURE 2:**
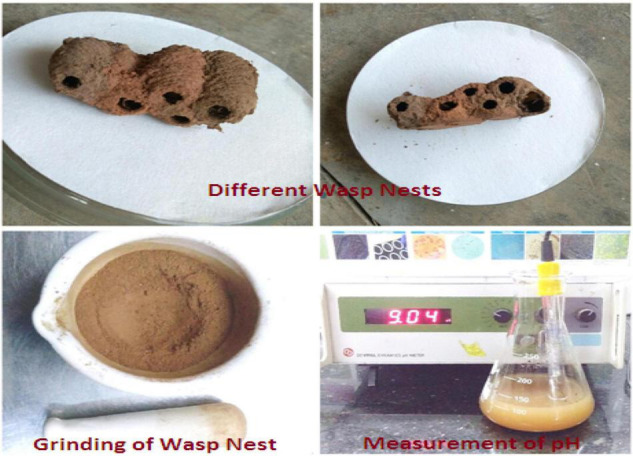
Collection of wasp nest and grinding of soil and pH measurement.

During the study, a total of 10 fungal species under 7 genera were obtained from wasp nest soil samples. Among the 10 species, 8 spp were identified under 5 fungal genera, and 2 were sterile mycelia (yellow and brown). *Paecilomyces variotii* was isolated from the mixed culture of wasp nest soil, whereas the fungus, *Paecilomyces variotii* was isolated from the normal ground soil for control (Figures 1A,B). Collected wasp nest and prepared soil by mortar and pestle with its alkaline pH value (9.4) are given in Figure 2. Both the soils and wasp nest soil isolate, *Paecilomyces variotii* culture filtrates, and aqueous filtrates were taken for the bioproduction of AgNPs and Ag^+^.

### Synthesized Nanoparticles

**FIGURE 3:**
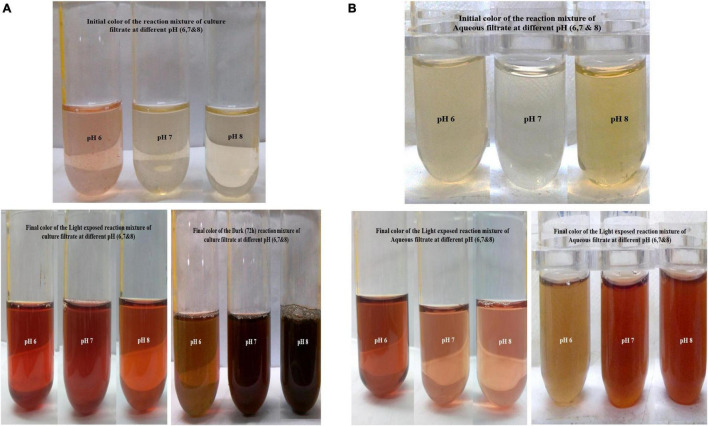
**(A)** Culture filtrate at different pH and sunlight-mediated biosynthesis of silver nanoparticles (AgNPs). **(B)** Aqueous filtrate at different pH and dark-mediated biosynthesis of AgNPs.

The flask containing reaction mixtures turned their initial pale yellow color to dark brown, golden brown, and black color as a final color (Figures 3A,B and Table 1). The reaction mixture in the dark took 72 h to change its color, whereas the sunlight-exposed reaction mixture took only 5 min to reach its final color. To cross check the production and efficiency of silver nanoparticles, *Paecilomyces variotii* was also isolated from ground soil sample and allowed for synthesis of silver nanoparticles. Both culture filtrate and aqueous filtrate of *P. variotii* of different habitats were compared. In the reaction mixture, it was clearly indicated by the appearance of final dark brown and golden brown color in the solution as the formation of silver nanoparticles.

**TABLE 1:** Biosynthesis of silver nanoparticle (AgNP) initial and final color of the reaction mixtures.

Reaction mixtures	pH value	Initial color	Final color	
			Light exposed	Dark
Reaction mixture 1 (AgNO_3_ + culture filtrate)	pH 6	Pale yellow	Brown	Brown
	pH 7	Pale yellow	Dark brown	Black
	pH 8	Dark yellow	Brown	Black
Reaction mixture 2 (AgNO_3_ + aqueous filtrate)	pH 6	Pale yellow	Brown	Golden yellow
	pH 7	Pale yellow	Brown	Dark brown
	pH 8	Pale yellow	Brown	Dark brown

**FIGURE 4:**
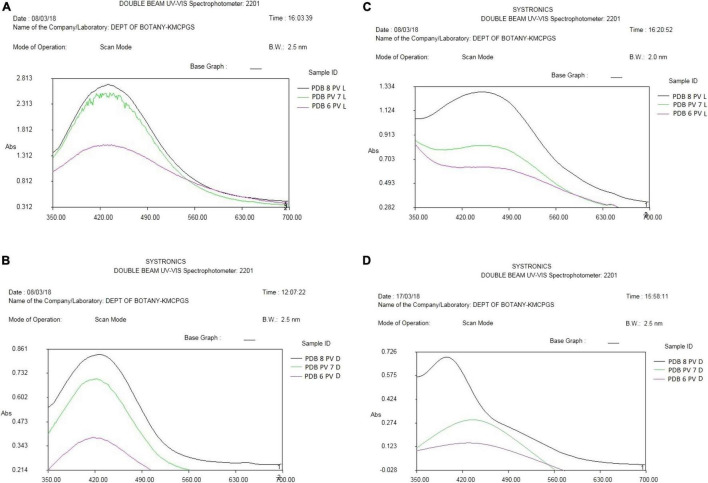
**(A)** UV-Vis (visible) spectroscopy of AgNP reaction mixture 1 of *P. variotii* light exposed. **(B)** UV-Vis spectroscopy of AgNP reaction mixture 1 of *P. variotii* dark sample. **(C)** UV-visible spectroscopy of AgNP reaction mixture 2 of *P. variotii* light exposed. **(D)** UV-visible spectroscopy of AgNP reaction mixture 2 of *P. variotii* dark-kept sample.

Synthesized silver nanoparticles were observed visually by altering their initial color from yellow to dark brown and golden brown, which were further confirmed by instrumental analysis such as UV-Vis spectrophotomer. The occurrence of color alteration may be due to the excitation of surface plasmon resonance in the silver metal nanoparticles (Nayak et al., 2013, Nayak and Anitha, 2014; Zarina and Nanda, 2014a; Nayak et al., 2018b). Among the different pH, pH 6 was found to be good for synthesis during light-mediated and dark condition, whereas pH 7 and 8 took more time for the conversion of the color. The synthesized AgNPs in the reaction mixture of culture filtrate at different pH range showed the maximum absorbance at 420 to 444 nm at different light conditions. pH 6 of light-exposed sample from the reaction mixture 1 (Table 2) gave the peak at 420 nm followed by 72 h dark condition gave a peak at 422 nm. pH 7 and pH 8 of both light-exposed and dark condition samples gave the peaks from 428 to 448 nm (Table 2), whereas the reaction mixture 2 showed the absorbance peak of the pH 6 light-exposed sample at 410 and 405 nm but dark condition sample showed 435 nm. pH-7 of light exposed, dark kept samples showed the peaks at 432 nm and 435 nm, respectively. Reaction mixture 2 of pH-8 of light exposed and dark condition samples showed the peaks at 448 and 444, respectively (Figures 4A–D). Previous work reported that the same surface plasmon peaks were at 420 nm by the AgNPs of *Aspergillus ochraceus.* Extracellular biosynthesis of AgNPs from aqueous filtrate of filamentous fungi was reported by Ahmed Rather et al. (2021), who explained that the silver nanoparticles synthesized by the filamentous fungi showed the maximum absorbance at 420–440 and 455 nm on a UV spectrophotometer. A few others reported that the surface plasmon absorbance peaks range were about 412–440 nm (Keller, 2004; Devika et al., 2012). The presence of plasmon band at 420 nm due to dipole plasmon resonance showing that the AgNPs have a spherical shape is reported by different researchers (Nayak et al., 2018a; Muthukrishnan et al., 2019a). The particular mechanism for the biosynthesis of silver nanoparticles has not been clear yet, but it has been recommended that the fungal biomass contains reducing agents, such as NADH-dependent nitrate reductase enzyme, the nitrate reductase secreted by the fungal cells (Devi and Joshi, 2012; Nanda et al., 2018). Another study reports that the influence of different pHs on the reaction media showed high efficacy for silver nanoparticle formation as well as their stability (Muthukrishnan et al., 2019a). They (Nayak et al., 2018b; Muthukrishnan et al., 2019b) also found out that pH 4 to pH 7 have strong support in the stable formation of silver and gold nanoparticles.

**TABLE 2:** UV-Vis (visible) spectrophotometric peaks at different light conditions.

Reaction mixtures	AgNO_3_ concentration	pH value	UV-Vis spectrophotometric peak	
			at different light conditions	
			Light exposed (5 min)	Dark (72 h)
Reaction mixture 1 (AgNO_3_ + culture filtrate)	1 mM	pH 6	420	422
		pH 7	428	428
		pH 8	428	428
Reaction mixture 2 (AgNO_3_ + aqueous filtrate)	1 mM	pH 6	410	405
		pH 7	432	435
		pH 8	448	444

### Antimicrobial Assay of Silver Nanoparticles

**FIGURE 5:**
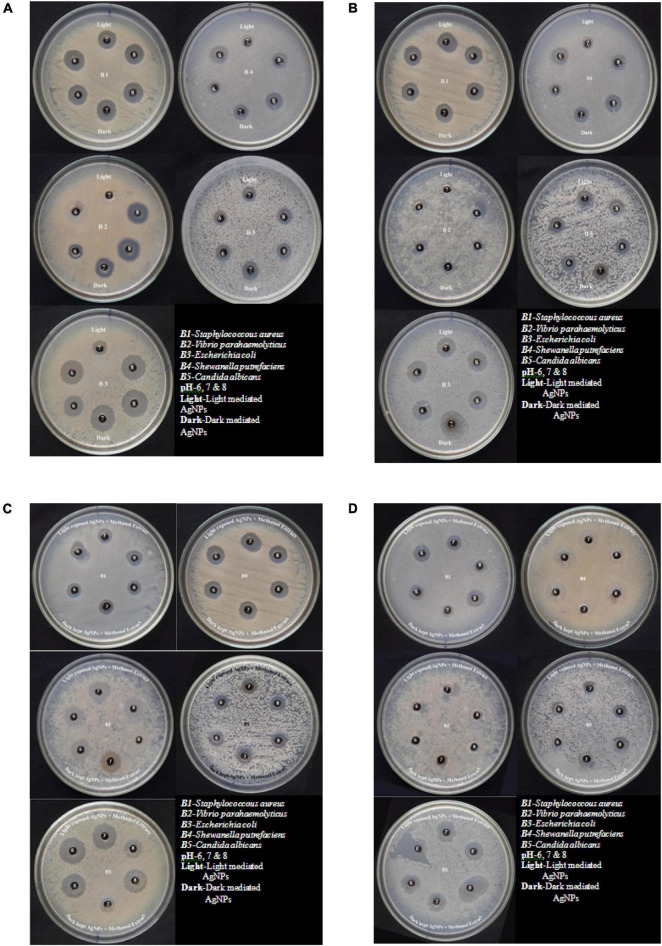
**(A)** Antimicrobial effect of light-mediated AgNPs synthesized from culture filtrate of *P. variotii*. **(B)** Antimicrobial effect of light-mediated AgNPs synthesized from aqueous filtrate of *P. variotii*. **(C)** Antimicrobial effect of light-mediated AgNPs synthesized from methanol extracted culture filtrate of *P. variotii*. **(D)** Antimicrobial effect of light-mediated AgNPs synthesized from methanol extracted aqueous filtrate of *P. variotii*.

The silver nanoparticles synthesized from the fungi of the habitats *viz*., wasp nest soil and ground soil, and at three pH were checked for their antimicrobial activity against different MTCC pathogenic bacteria and *Candida albicans*. The maximum-sized zone of inhibition was observed against *E. coli* (20 mm) at different light sources and pH 8 condition of culture filtrates (Figures 5A,B and Tables 3A,B, 4). Silver nanoparticles from *P. variotii* of culture filtrate pH 6 light-exposed samples, pH 7 and pH 8 dark 72-h samples were given the 16-mm diametric size of zone against *E. coli*, and it was followed by *Staphylococcus aureus* (19 mm), *Vibrio parahaemolyticus* (16 mm) at pH 8 light-exposed sample and dark condition samples, respectively. *Candida albicans* showed the maximum zone of inhibition (12 mm) at pH 7 light-exposed sample, and *Shewanella putrefaciens* showed the zone of inhibition (12 mm) at pH 8 dark condition. During the antimicrobial study, notable significant difference was found in inhibitory zones on sunlight-exposed and dark condition samples at all the pH ranges.

**TABLE 3A:** Antimicrobial activity of cultured filtrate and different light condition-mediated biosynthesized AgNPs.

Pathogens	Reaction mixture 1 (AgNO_3_ + culture filtrate)					
	pH 6		pH 7		pH 8	
	L	D	L	D	L	D
*Staphylococcus aureus* (B1)	15	14	13	13	19	14
*Vibrio parahaemolyticus* (B2)	–	10	–	13	15	16
*Escherichia coli* (B3)	16	13	10	19	20	20
*Shewanella putrefaciens* (B4)	13	08	08	10	10	12
*Candida albicans* (B5)	10	11	10	12	10	09

**TABLE 3B:** Antimicrobial activity of aqueous filtrate and different light condition-mediated biosynthesized AgNPs.

Pathogens	Reaction mixture 2 (AgNO3 + Aqueous filtrate)					
	pH 6		pH 7		pH 8	
	L	D	L	D	L	D
*Staphylococcus aureus* (B1)	13	14	16	13	13	13
*Vibrio parahaemolyticus* (B2)	–	10	–	–	10	–
*Escherichia coli* (B3)	15	13	13	18	14	13
*Shewanella putrefaciens* (B4)	12	08	08	10	10	12
*Candida albicans* (B5)	10	10	08	10	10	10

**TABLE 4:** Antimicrobial activity of biosynthesized AgNPs combined with methanol extract of culture filtrate.

Pathogens	AgNPs of culture filtrate + methanol extract of culture filtrate						AgNPs of aqueous filtrate + methanol extract of culture filtrate					
	pH 6		pH 7		pH 8		pH 6		pH 7		pH 8	
	L	D	L	D	L	D	L	D	L	D	L	D
*Staphylococcus aureus* (B1)	13	10	10	10	11	10	12	10	10	–	–	11
*Vibrio parahaemolyticus* (B2)	14	13	15	13	13	10	8	8	8	8	8	8
*Escherichia coli* (B3)	17	15	15	–	11	15	20	14	13	13	13	16
*Shewanella putrefaciens* (B4)	11	11	13	10	11	12	10	8	8	10	10	10
*Candida albicans* (B5)	10	10	10	8	10	10	10	10	10	10	10	10

Silver nanoparticles synthesized from *Paecilomyces variotii*; reaction mixture 2 also showed the notable zone of inhibitions. The maximum zone of inhibition was observed against *E. coli* (18 mm) at pH 7 dark condition sample, and it was followed by *Staphylococcus aureus* (16 mm) at pH 7 light-exposed samples. AgNPs of pH 6 and pH 8 dark sample inhibit the growth of *Vibrio parahaemolyticus* at 10-mm zone of inhibition. *Candida albicans* showed the average size of zone (8 mm). The maximum zone of inhibition was observed in both light-exposed and dark condition AgNPs of all pH ranges against all pathogenic bacteria and fungus (Nayak and Nanda, 2014a).

**FIGURE 6:**
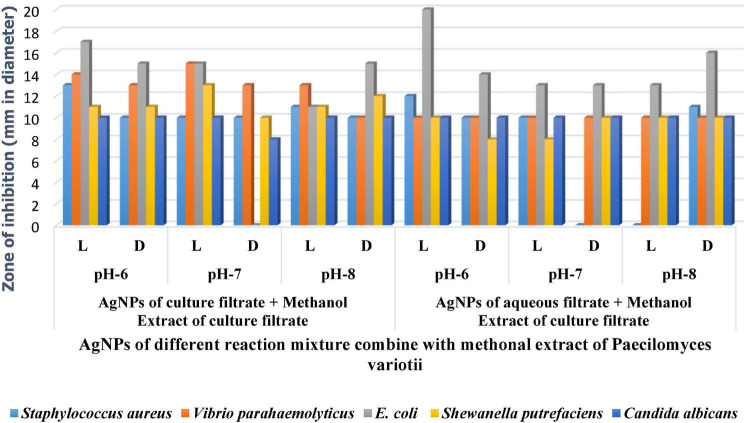
Antimicrobial activity of AgNP of *P. variotii* combined with methanol extract.

The synergetic effects of AgNPs combined with methanol extract (bioactive compounds) of *Paecilomyces variotii* culture filtrate were tested against bacteria and fungus (Figures 5C,D and Table 4). The maximum zone of inhibition (20 mm) was observed in reaction mixture 2 of pH 6 light-exposed sample against *E. coli*, and it was followed by *Vibrio parahaemolyticus* (15 mm) at light-exposed sample of pH 7 in reaction mixture 1. All the pathogenic test organisms were highly inhibited by the methanol extract-combined silver nanoparticles (Figure 6). The inhibition zone size of methanol extract-combined AgNPs were lesser, but their inhibiting stability was remarkably constant when compared with the methanol extract-non-combined AgNPs (Figures 5C,D).

### Particle Morphology and Size by Scanning Electron Microscopy

**FIGURE 7:**
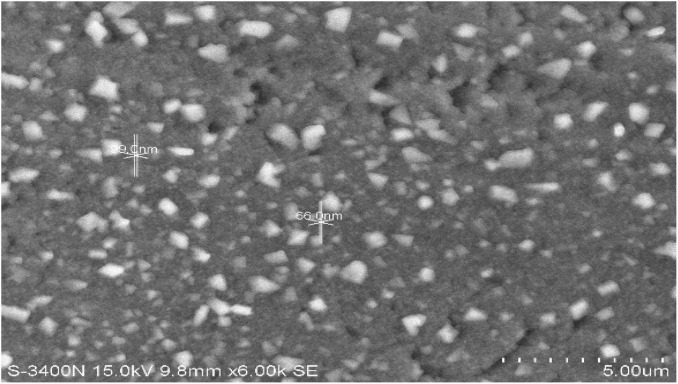
Scanning electron microscopy (SEM) image of AgNPs of *P. variotii.*

The size and shapes of the AgNPs were analyzed, and most of the particles of dark condition samples appeared polygonal with a broad size distribution form varying from 20 to 70 nm. Interestingly, light-exposed samples of all pH showed triangle-shaped particles at different sizes (15 to 90 nm). Rod-shaped particles were also found in all the samples. The SEM analysis revealed that the average particle size was approximately 38 nm (Figure 7).

### Fourier-Transform Infrared Analysis of Silver Nanoparticles

**FIGURE 8:**
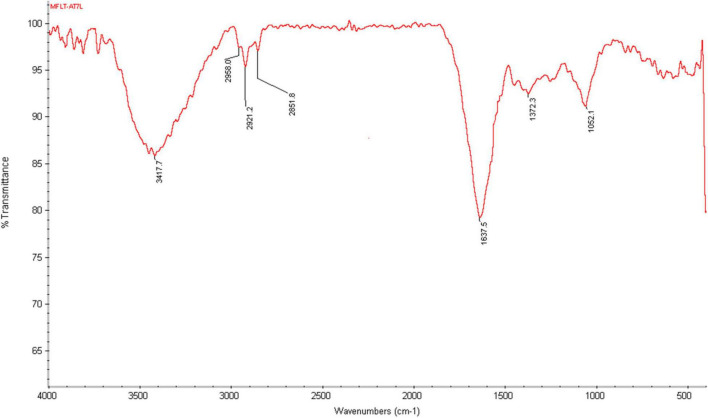
Characterization of AgNPs by Fourier-transform infrared (FTIR) of *P. variotii*.

Spectral measurement was carried out to identify the possible biomolecules responsible for efficient reduction of silver ions (Ag^+^) and capping agents (proteins) for the stabilization of the resultant silver nanoparticles (AgNPs), which occurred through the participation of fungal culture filtrate proteins (Figure 8). FTIR spectra of AgNPs synthesized from *Paecilomyces variotii* light-exposed sample showed the absorption bands at 3,439.3, 1,622.0, 1,383.1, 1,028.6, 590.9, 561.5, 468.1, and 436.4, respectively, whereas dark condition sample showed bands at 3,449.0, 2,924.4, 2,851.8, 1,620.8, 1,383.8, 1,258.1, 1,033.5, 534.8, and 466.4 at different transmittance (Figure 8). The functional groups such as C–H stretch, C–O–C, CH_3_–R, N–H, aromatic C–C skeletal vibrations, –NO_3_, COO, thioester, and S–S stretch were observed at different wave numbers. The previous studies reported that functional groups, such as –C–O–C–, –C = C−, and –COO, are derived from heterocyclic compounds, such as proteins present in the fungal extract and are capping ligands of AgNPs (Zarina and Nanda, 2014b). The bands at 3,422 cm^–1^ in the spectra correspond to O–H stretching vibration indicating the presence of alcohol and phenol. Bands at the 2,921- and 2,856-cm^–1^ region arising from C–H stretching of aromatic compounds were observed. The band at 1,631 cm^–1^ in the spectra corresponds to C–N and C–C stretching indicating the presence of proteins (Zarina and Nanda, 2014b). The band at 1,450 cm^–1^ was assigned for N–H stretch vibration present in the amide linkages of the proteins. These functional groups have a role in stability/capping of AgNP as reported in many studies (Zarina and Nanda, 2014b). The bands at 1,383 and 1,043 cm^–1^ were assigned for N–H and C–N (amines) stretch vibration of the proteins, respectively. The band at 1,258 cm^–1^ corresponds to C–N stretching of amines. The bands at the 590-cm^–1^ region could be attributed to C–Br stretching, which has the characteristic of alkyl halides. FTIR studies confirmed that the carbonyl groups of amino acids and peptides of proteins have a strong affinity to bind metal ions, and they may encapsulate nanoparticles leading to their stabilization in the AgNPs synthesized from *Paecilomyces variotii* in our study, which was confirmed by earlier reports (Johnson, 2014).

### X-ray Diffraction Analysis of Silver Nanoparticles

**FIGURE 9:**
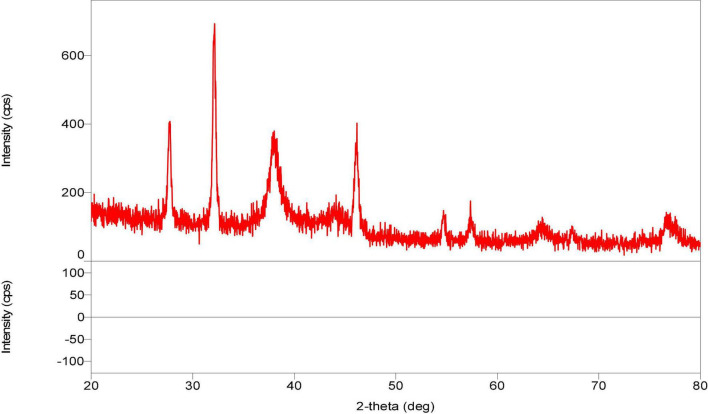
X-ray diffraction (XRD) analysis of silver nanoparticles synthesized from *P. variotii*.

These biological silver nanoparticles were further characterized by x-ray diffraction (XRD) technique used to determine the metallic nature of nanoparticles. X rays are actually electromagnetic radiations with photon energy in the range of 100 eV–100 KeV. These highly energetic x rays, which have high penetration power that enters deep into the material and provides detailed information about the material. The nanoparticles of *Paecilomyces variotii* showed the XRD peak values at 38, 44, 54, 57, 64, and 76, respectively (Figure 9). The current results agreed with the previous studies made by the following workers (Saravanan and Nanda, 2010; Nanda and Majeed, 2013; Muthukrishnan et al., 2019b; Majeed et al., 2021).

The same work as ours was reported by Honary et al. (2013) who explained that FTIR helps in identifying the possible biomolecules responsible for capping and efficient stabilization of the metal nanoparticles synthesized by *Penicillium citrinum*. They also observed that the FTIR spectra have different functional groups, such as amide linkages and – COO – which are probably sandwiched between amino acid residues in the protein and the synthesized silver nanoparticles (Sathishkumar et al., 2009; Saravanan and Nanda, 2010; Honary et al., 2013).

## Conclusion

During our present study, the wasp nest soil was observed to be an enriched source of microbial diversity of numerous fungi. The extract and its biosynthesized silver nanoparticles of the wasp nest fungal isolate, *Paecilomyces variotii*, were found to be as good as antimicrobial compounds for the prevention of MTCC pathogens. pH 6 was found as the best pH for the synthesis of silver nanoparticles in our present study in comparison with 7 and 8 pH. SEM results were confined to different shapes and sizes of the silver nanoparticles along with UV-VIS absorption spectra. FTIR analysis showed the distinction of functional groups in the AgNPs independently in *Paecilomyces variotii*. XRD analysis confirmed the metallic nature and size of the silver nanoparticles. The findings of our study in favor of the potential role of *Paecilomyces variotii* may prove that the experimental support that insect-associated microbes are a valuable source for natural products and can be applied in designing noble nanomaterials to prevent pathogenic bacteria in order to control human pathogens in the near future.

## Data Availability Statement

The original contributions presented in the study are included in the article/supplementary material, further inquiries can be directed to the corresponding author.

## Author Contributions

The authors are the supervisor and the research scholars of a team working toward the potential nanomaterials for Drug resistant pathogens. All authors contributed to the article and approved the submitted version.

## Conflict of Interest

The authors declare that the research was conducted in the absence of any commercial or financial relationships that could be construed as a potential conflict of interest.

## Publisher’s Note

All claims expressed in this article are solely those of the authors and do not necessarily represent those of their affiliated organizations, or those of the publisher, the editors and the reviewers. Any product that may be evaluated in this article, or claim that may be made by its manufacturer, is not guaranteed or endorsed by the publisher.
